# Laboratory medicine between technological innovation, rights safeguarding, and patient safety: A bioethical perspective

**DOI:** 10.1515/med-2025-1153

**Published:** 2025-05-29

**Authors:** Francesco De Micco, Gianmarco Di Palma, Flavia Giacomobono, Anna De Benedictis, Mariano Cingolani, Vittoradolfo Tambone, Luca Tomassini, Roberto Scendoni

**Affiliations:** Research Unit of Bioethics and Humanities, Department of Medicine and Surgery, Università Campus Bio-Medico di Roma, Roma, Italy; Operative Research Unit of Department of Clinical Affairs, Fondazione Policlinico Universitario Campus Bio-Medico, Roma, Italy; Research Unit of Nursing Science, Department of Medicine and Surgery, Università Campus Bio-Medico di Roma, Roma, Italy; Department of Law, Institute of Legal Medicine, University of Macerata, Macerata, Italy; International School of Advanced Studies, University of Camerino, Camerino , Italy

**Keywords:** laboratory medicine, diagnostic and therapeutic evolution, ethical concerns, patient data privacy, healthcare equity, healthcare integration, personalist bioethics

## Abstract

**Introduction:**

The evolution of Laboratory Medicine (LM) has expanded diagnostic and therapeutic horizons, yet ethical concerns pervade its advancements. This article explores the ethical challenges inherent in LM and advocates for a personalist bioethical framework as a guiding principle. It addresses issues such as patient data privacy, healthcare equity, communication of complex analysis results, genetic information management, and conflicts of interest.

**Objectives:**

The proposed framework emphasizes principles such as the defense of life, freedom, responsibility, totality in therapy, and sociality and subsidiarity, with a focus on human dignity.

**Results:**

Various bioethical theories address complex ethical issues in medicine and health sciences, often combined to guide clinical practice. This study focuses on personalist bioethics, prioritizing human dignity and intrinsic value, advocating for principles like defense of physical life, freedom, totality in therapy, and sociality. In LM, ethical analysis involves patient care, genetic testing, informed consent, and Artificial Intelligence integration, emphasizing transparency and patient autonomy.

**Conclusion:**

LM plays a crucial role in healthcare, necessitating ethical considerations amidst technological progress. Upholding ethical frameworks, such as the ethics of good work, can ensure equitable and quality healthcare delivery.

## 1 Introduction

Laboratory medicine (LM) is a young science characterized by broad horizons and fascinating potential, synthesis between different branches such as Pathology, Clinical Biochemistry, Clinical Microbiology, and the contribution of new methodological perspectives appropriate to Medical Biotechnologies [1,2]. Its development has led to many significant innovations. New diagnostic technologies such as genomics, proteomics, and metabolomics have made a more precise and personalized diagnosis of diseases possible. These approaches allow the analysis of DNA, proteins, and metabolites to identify the causes and characteristics of diseases. Clinical analysis laboratories are becoming increasingly automated, reducing human errors, increasing efficiency, and allowing greater productivity. Robots are employed to perform repetitive and standardized analyses [3,4,5]. LM is moving toward more personalized approaches, taking into account the individual genetic and molecular characteristics of patients. This allows for the customization of treatments to maximize effectiveness and reduce side effects. Faster and portable diagnostic tests have been developed, allowing results to be obtained in minutes rather than hours or days. These tests are crucial for early diagnosis and disease control [6,7]. Information systems and big data play a crucial role in analyzing and interpreting laboratory results. The integration of this data allows for better management of patient information and contributes to evidence-based medicine [8,9]. Research continues to identify new biomarkers, which are biological indicators that can be measured and used to assess health status or the presence of diseases. These biomarkers often become targets for new therapies [10,11,12].

The evolution of LM has expanded diagnostic and therapeutic possibilities, enabling a better understanding of diseases and paving the way for more targeted and personalized treatments. However, it also presents some ethical issues that require attention.

With the increasingly widespread use of genomics and other advanced technologies, the collection and management of patient’s genetic and personal data become crucial. Ensuring data privacy and establishing secure protocols for handling sensitive information are important to prevent abuse or privacy violations [13,14]. New diagnostic technologies can be expensive and not always accessible to all patients, leading to disparities in access to healthcare. This raises ethical issues regarding fairness in the provision of healthcare services and ensuring that everyone can benefit from advances in LM [15]. The complexity of laboratory analysis results can make it difficult for patients to understand. Proper communication and interpretation of results are crucial to avoid misunderstandings or unnecessary anxieties. Genetic information can reveal predispositions to diseases or health conditions. For this reason, it would be advisable for the interpretation of the data and communication to the patient to be handled by Medical Laboratory Professionals (MLPs) with specific expertise in the field of genetics. This is to effectively convey to the patient the implications of the diagnosis in a clear and realistic manner while allowing the patient to actively participate in decisions regarding their health, respecting their dignity and autonomy. Furthermore, the use of such information raises ethical questions about genetic discrimination, stigmatization, and the management of information by insurance companies or employers [15,16,17].

Finally, in research laboratories and companies developing diagnostic technologies, conflicts of interest may arise between pursuing profit and the public interest in health. This could influence the presentation of data or the promotion of unnecessary tests or treatments [18,19].

The purpose of the article is to highlight the ethical issues related to the professional practice of LM and to propose an ethical framework as a reference to address these challenges and pursue safe and quality LM.

## 2 Bioethical framework

Multiple bioethical theories are available to reference, each offering a unique perspective in addressing complex ethical issues within medicine and health sciences, often used in combination to guide ethical decisions and clinical practice [20]. The bioethical framework referred to in this work is that of personalist bioethics [21,22]. It is an ethical approach that places human dignity and intrinsic value at its core. It focuses on the individual as a subject endowed with dignity and inalienable rights, aiming to protect and promote human well-being and dignity in every biomedical context. Conversely, the principialist and consequentialist models were not deemed suitable for the present analysis. While principlism offers a well-structured approach, it may not fully capture the relational aspects of patient care. On the other hand, consequentialism, with its outcome-oriented perspective, adopts a highly pragmatic approach but risks overlooking individual dignity [23]. This personalistic approach is based on several fundamental principles:

a) *The principle of defense of physical life.* The principle of defense of physical life is one of the fundamental principles in bioethics, referring to the moral duty to protect and preserve human life. This principle underscores the intrinsic value of life and the ethical obligation to do everything possible to avoid physical harm or dangers that may compromise the health and physical well-being of individuals. From this principle arises the ethical imperative of respecting human life, wherein the defense and promotion of life have a limit in death, which is a part of life, and the promotion of health has a limit in illness, which should be healed and treated, and in any case considered with an active approach, even when it is incurable. In a laboratory context, this principle requires quality control measures to be taken in order to avoid errors in diagnostic results that could pose a danger to the patient. For example, ensuring analytical accuracy to prevent errors in diagnosis.
b) *The principle of freedom and responsibility*. This principle refers to people’s capacity to act according to their own will and to take responsibility for their actions, decisions, and consequences. Freedom is closely linked to the concept of autonomy. It acknowledges the right and ability of individuals to make autonomous decisions regarding their own lives, health, and medical treatments. Freedom implies respect for individual rights, including rights such as privacy, freedom of expression, and the right to religious freedom. These rights can influence medical decisions, such as the choice of treatments based on personal or religious beliefs. However, the physician has the freedom to dissent from any requests that conflict with their conscience that are contrary to legal norms, professional ethics, or good clinical care practices. Freedom is reflected in the principle of informed consent, which ensures that patients are fully informed and free to accept or refuse medical treatment based on detailed information. In the laboratory setting, patients should be informed about how their genetic data might be used, with the option to withdraw their consent at any time.
c) *The principle of totality or therapeutic principle*. Intervention in a person’s physical life is lawful where it is necessary for the safeguarding of the person’s own life. Therefore, it is necessary that when administering therapy, it is evaluated within the entirety of the person, demanding a certain proportion between the risks and damages it may entail and the benefits it provides. Administering disproportionate treatments without foreseeable results may demonstrate aggressiveness or therapeutic obstinacy. In such a case, within a laboratory, Artificial Intelligence (AI) systems could provide in-depth analyses of diagnostic results. However, the physician should always contextualize and validate this information, ensuring the comprehensive management of the data.
d) *The principle of sociality and subsidiarity*. According to this principle, the common good is achievable through the good of the individual and solidarity toward those in greater need. Each citizen must consider not only their own life but also that of others as a personal and social good. The community, in turn, commits to promoting the life and health of everyone, ensuring that all have access to medical care and providing more assistance where the need is greatest [21,22]. In such a case, in keeping with the principle, it could be beneficial to introduce mobile laboratories or subsidies for screening in underserved areas.

Personalistic bioethics, thanks to its focus on the concept of human dignity and the recognition of the intrinsic value of the person, can find concrete applications in various areas of LM. In particular, in the delicate area of genetic testing, this perspective requires that the patient be informed about the potential results, often of a probabilistic nature, as well as about the possible impacts that such analyses may have on the life of the subject. In fact, by not providing unequivocally certain results regarding the materialization of a possible pathology, such analyses may influence a person’s life choices to a variable extent. This is essential to fully guarantee respect for the right to decisional autonomy. Similarly, in the use of laboratory diagnostic tools based on AI, personalist bioethics emphasizes the importance of a correct balance between technological efficiency and respect for the person. As is known, one of the major technical problems related to the use of AI is the lack of transparency in the algorithmic evaluation process. An inadequate understanding of the functioning of these systems represents a violation of the patient’s right to self-determination. In this sense, the personalist approach requires the implementation of approaches capable of increasing the explainability of AI systems, involving the patient in the understanding and management of the results.

## 3 Bioethical analysis

MLPs operate within three major areas: patients, medical colleagues, and other professional figures they engage with, as well as society itself [24,25]. It is precisely within this broad spectrum of work that ethical reflection becomes necessary. The urgent need to provide scientifically updated and clinically valid responses obliges MLPs to ensure that every phase of the processes they are responsible for maintains high standards in terms of quality, effectiveness, and safety. Only by doing so can patients be assured of receiving the best possible care, aligning with a modern and increasingly essential rationalization of resources.

### 3.1 The patient

As with any branch of medical science, the core of action in LM is centered around the patient. The current capability to perform and report laboratory tests in real-time, especially in urgent and emergency medicine, has altered the approach to patient care and its clinical management. The expertise within a medical laboratory holds direct responsibility for the quality and integrity of the service provided. This necessitates maintaining high standards of competence to safeguard patients from incompetent or even illegal practices.

A primary aspect concerns the vast amount of data a laboratory possesses to adequately identify patients. Laboratories must comply with data privacy laws and regulations, such as the General Data Protection Regulation (GDPR) in Europe or the Health Insurance Portability and Accountability Act (HIPAA) in the United States [26,27,28,29,30]. Therefore, ensuring patient information confidentiality and safeguarding personal data from unauthorized access and improper use is crucial. Rigorous cybersecurity standards are necessary to protect sensitive data, employing encryption, restricted access, firewalls, and other protective measures to prevent unauthorized access or intrusion [14,31,32].

Furthermore, patients should be properly informed about the type of information stored and the intended use (clinical, research, educational, etc.). Patients must provide informed consent for the collection and processing of data and should have some level of control over the use of their data, including the ability to revoke consent or limit access to specific information [33,34].

In this regard, it is worth highlighting the role of laboratories in biomedical research and the need for professionals involved to identify potential benefits and risks, informing patients about the procedures adopted, the risks, benefits, side effects, and possible alternative diagnostics [34,35,36,37]. Regarding procedures performed by MLPs, more invasive procedures necessitate adequate informed consent, whereas routine procedures like a venous blood draw can be implied when a patient spontaneously requests professional service. In this regard, two issues are particularly interesting for their ethical implications: Direct-to-Patient Report delivery and Direct-to-Consumer laboratory testing (DTC). In both cases, the interpretation of laboratory data may not be performed by a physician, with significant diagnostic and therapeutic consequences. Yet, the relational dimension and the need to inform the patient play a fundamental role in the field of medicine, given the specific contribution of medicine to the study of humans in a constitutive dimension, such as illness. An impersonal approach or even the absence of a relationship reduces, even nullifies, the space and time for care, limiting the autonomy of the patient as they are poorly informed or not informed at all. Therefore, laboratories should always provide assistance to their patients, even when tests are not directly under their responsibility, as in the case of DTC, also by establishing dedicated communication teams and developing, implementing, and verifying a communication plan [38]. In this way, the principles of defending physical life, freedom, and responsibility would be pursued, even for those segments of the population who are economically disadvantaged and can meet their health needs by accessing low-cost laboratory tests, without resulting in an informational deficit with consequences on their health status [39].

A separate consideration is warranted for genetic tests. Genetic information is highly sensitive and can reveal intimate health-related data and predisposition to certain diseases. For example, a patient’s genetic test results might be accidentally sent to an unauthorized recipient, exposing the patient to potential insurance discrimination. Genetic test results can have a strong emotional and psychological impact on the patient and their family. Discovering predispositions to serious illnesses can generate anxiety, stress, and concerns for the future, considering these data could be used for discrimination in various contexts, such as insurance, employment, or access to services [40,41,42,43,44]. Therefore, it is essential to ensure that patients are fully informed about the implications of genetic tests and provide their informed consent before undergoing such tests. Hence, a pre-test counseling session is mandatory to educate patients about the implications of these tests, assist them in understanding and managing the test results, and provide resources to address psychological implications [45,46,47]. For instance, a patient using a genetic testing kit without professional counseling might experience anxiety and misinterpretation of the results.

In assessing patient safety concerns related to digitization and automation, it is essential to consider the enhancement of LM through AI. AI will increasingly integrate with LM, enhancing diagnostic capabilities but also raising associated risks. It is conceivable that an AI system implemented for laboratory analysis could erroneously classify an autoimmune disease as a viral infection, leading to inappropriate treatments. This aspect underscores the principle of totality, which requires a balance between the use of technological tools and human critical intervention to ensure accurate clinical decisions [48]. The latest machine learning models act like “black boxes,” having a structure so complex that users cannot comprehend how an AI system converts data into decisions [49]. Uncontrolled and erroneous decision-making by an algorithm can cause severe and irreversible damage. The growing integration of AI in LM demands absolute transparency between doctors and patients, among doctors and healthcare organizations, and between healthcare organizations and the public, as it is essential for quality, safety, accountability, and informed decision-making [50]. The opacity of algorithmic decisions, autonomous behavior, and the complexity of AI systems make it extremely difficult for patients to prove liability in cases of harm resulting from medical errors. This can lead to prolonged timelines and, consequently, higher costs for legal proceedings. Furthermore, it should be noted that the unregulated use of AI could exacerbate inequalities in access to care, particularly if the system’s design fails to adequately consider the needs of the most vulnerable populations [51].

According to Pennestrì and Banfi, patients should explicitly consent to the use of their health data for purposes other than their health and be aware that medical decisions regarding their health are based on data processed by machines. Otherwise, there is a risk of breaking a fundamental element of the doctor-patient relationship, which is trust [52]. The concrete risk is the impoverishment of the perception of reality, which would be reduced to a formal datum, potentially leading to a crisis of identity and a loss of sense of one’s work activity. As Heidegger states, we need meditative thinking capable of confronting us with the complete dominion of technology [53].

The principle of defending physical life requires MLPs to be aware of the potential risks associated with the manipulation of biological samples when pervasive use of new technologies is employed. Therefore, it is necessary to ensure that all activities are conducted with utmost care and consideration for the health and physical well-being of patients, including fields such as patient privacy protection and data security, to prevent any unauthorized exposure or breach of confidentiality. The two main privacy regulatory models are the GDPR in Europe and the HIPAA in the United States. These regulatory frameworks are essential tools for protecting personal data, including health information, and are a cornerstone of privacy and security management in healthcare practices. They establish clear rules to ensure that data processing is lawful, transparent, and secure while safeguarding patient rights. The personalist approach aligns well with the principles established by the GDPR and HIPAA, as both models prioritize the protection of privacy and respect for individual rights. Like personalist ethics, these two regulatory frameworks place great emphasis on informed consent and patient autonomy. For example, in LM, this means that patients must be aware that their biological samples and related data are being used for diagnostic and therapeutic analysis. Another fundamental principle reflected in both regulatory frameworks is respect for dignity and privacy. In LM, it is essential to ensure that patient information, including genetic analyses and other sensitive data, is protected from any form of misuse or unauthorized disclosure. Furthermore, both models emphasize the concepts of personalization of treatment, transparency, and accountability. These aspects converge with the personalist model, which requires that data be processed for specific and targeted purposes while ensuring responsible use to promote trust in the health system. Therefore, it is evident that these regulations provide a solid basis for integrating personalist ethics, ensuring that each patient is treated with the utmost respect and that their information is used exclusively for their well-being [54,55,56].

The results of a laboratory test, like access to medical data, should be solely accessible to the clinician who requested the examination, the patient, or those the patient has authorized according to legal standards, the laboratory staff, or the healthcare facility if necessary within the patient’s diagnostic and treatment process. In any case, if data are shared with other healthcare providers or entities, it is important to do so securely and protected through secure networks and encrypted protocols [57,58,59].

It will also be necessary for patients to be properly and comprehensively informed that data concerning their health may be processed by machines without the interpretation of the data by a doctor. If this information is not provided, the principle of freedom and responsibility, which emphasizes the importance of balancing patients’ right to make autonomous decisions about their health with the responsibility of MLPs to provide them with informative and compassionate support during this process, would be violated.

AI being able to analyze huge amounts of clinical data allows the integration of information from different sources, helping to identify patterns and correlations that are difficult to detect manually [60]. A hybrid clinical methodology in which the human being’s critical thinking, i.e., the ability to analyze the available facts, evidence, observations, and arguments to form a judgment, is the apex of decision-making, is an extrusion of the principle of wholeness in that it emphasizes the importance of considering the whole individual, rather than treating only symptoms or illnesses in isolation, to provide effective and personalized treatment. Finally, it is imperative that, as in other fields of medicine, AI does not become a source of injustice and inequality. Indeed, AI can provide faster and more efficient diagnoses, but if these technologies are not distributed equitably or are not accessible to all socio-economic groups, it could increase inequalities in access to care precisely at the expense of those who need it most [61,62].

In such a scenario, while AI ensures improved efficiency and diagnostic precision, it remains essential to preserve the human element to guarantee patient-focused care. Automation can simplify diagnostic processes but cannot replace the professional in the empathetic communication of results. This aspect ensures respect for the principle of freedom and responsibility, enabling patients to make appropriately informed decisions about their health. AI algorithms should represent a supporting tool, not a substitute for the clinician’s judgment. A hybrid approach should be promoted, where AI provides in-depth analysis while the human operator contextualizes and validates the data. This approach embodies the principle of totality, emphasizing the consideration of the person as a whole rather than isolated technological data. Thus, in the context of technological advancements driven by AI, obtaining informed consent on how data are processed and interpreted becomes essential. Patients should be informed not only about the purpose and functioning of diagnostic tests but also about the specific role AI plays in the procedure, including its potential benefits, limitations, and possible biases. Communication should avoid technical jargon, and pre- and post-test counseling sessions should be provided. For example, explaining the difference between a probabilistic result generated by AI and a genetic predisposition is crucial, as this distinction could significantly influence the patient’s health decisions [63].

Enhancing patient autonomy and awareness could also be achieved through the implementation of dynamic consent models. These models allow patients to progressively update their consent preferences as personal data accumulates and algorithms evolve. Additionally, promoting educational campaigns – through brochures, online platforms, or television initiatives – could further enhance understanding and demystify AI-based diagnostics.

Moreover, the use of direct-to-consumer tests, as discussed, could undermine the relational dimension with the patient. To prevent this, laboratories could establish communication teams for patients using such tests. The issue of the gap between technological advancements and empathy could be addressed by ensuring enhanced ethical training for professionals and fostering relational skills. It is also crucial to focus on the digital divide. A potential solution could involve promoting mobile laboratory units or subsidies for screening tests in disadvantaged areas. Such an approach would ensure full compliance with the principle of subsidiarity [64,65].

As seen so far, the integration of AI in LM can represent a significant breakthrough, but it raises complex ethical issues. For example, the use of specific biomarkers can improve the accuracy of the diagnostic process, at the same time, if the algorithm’s training datasets are not representative of all populations, the system could autonomously perpetuate errors. An example could be a cardiovascular risk prediction algorithm, also based on laboratory variables, which, trained on a predominantly Caucasian population, could commit errors of underestimation or overestimation if applied to different ethnic groups. To mitigate these risks, as already analyzed, it is essential to implement technologies capable of ensuring transparency in decision-making models and developing human control mechanisms capable of balancing the reliability of technological tools and clinical judgment.

### 3.2 LM within the context of medicine

LM is a crucial pillar in modern medical practice, providing vital support for the diagnosis, treatment, and monitoring of health conditions. Collaboration between diagnostic laboratories and other sectors of medicine is essential to ensure comprehensive and high-quality care for patients.

Medical acts are integrated processes involving various actors with diverse skills, all focused on the patient’s well-being. The convergence of these actors toward a singular goal – the patient’s health – qualifies the medical act as genuinely integrated. Evaluating the integration of these actors’ partial objectives in their actions becomes crucial. The ethical goodness of a medical act is also judged by how well it is performed, objectively evaluating its execution. The necessary conditions for an ethically good medical act are its proper execution, adherence to the best evidence, compliance with the gold standard, and correct nosographic framing [66].

MLPs should uphold the dignity and respect of their profession as an integral part of the medical act. This can be achieved by significantly contributing to the advancement of their field, adhering to the ethical imperative of continuous professional development through a modern lifelong learning approach, promoting high standards of both theoretical and practical learning, and collaborating with other healthcare professionals.

This significantly impacts diagnosis and disease treatment, research and development, and prevention. LM, beyond providing tests for accurate diagnoses and aiding clinicians in determining the most suitable treatments, can identify specific biomarkers guiding personalized therapies, enabling doctors to tailor treatments based on individual patient characteristics [67]. In the latter case, laboratories can support the assessment of innovative therapies by monitoring patient responses to experimental treatments through data analysis [68,69].

Moreover, using laboratory tests for disease screening allows for early diagnosis, preventive interventions, monitoring of disease progression, and assessing the long-term effectiveness of treatments [70].

### 3.3 LM within the social context

LM, like other branches of Medical Services, offers a transparent, measurable, and robust cost analysis. This makes it particularly susceptible to external scrutiny through analysis methods and subsequent measures that mostly consider quantitative aspects.

The relationship between LM and business ethics is significant as clinical laboratories operate in a commercial context while providing essential services in the healthcare field.

It is crucial for laboratories to ensure cost transparency of tests and services to avoid financial surprises for patients. This guarantees fair access to laboratory services, reducing disparities in access to diagnostic tests and treatments [71]. It could be the case of an automated screening study that, when conducted in an underprivileged region, improves diagnostic accuracy. However, the lack of access to laboratories excludes low-income patients.

Furthermore, the corporate social responsibility of LMs is significant, involving participation in initiatives supporting public health and community well-being. This includes awareness programs, screenings, or support for local healthcare initiatives, as well as adopting sustainable and responsible practices to reduce the environmental impact of laboratory activities [71,72].

LMs can play a significant role in a health perspective based on a One Health approach. One Health is an integrated, unifying approach that aims to sustainably balance and optimize the health of people, animals, and ecosystems [73]. The impact of LM can be decisive in identifying emerging and re-emerging infectious diseases, implementing more effective control measures to combat zoonotic diseases, and improving public health through food safety [74]. In this sense, LM could contribute to the recovery of a genuine conception of bioethics, namely a research tool that encompasses human life, animal life, and the environment in its scope of action in order to create a bridge between the human and biological sciences for human survival [75]. This perspective highlights the fundamental ethical value of the work of LMPs, which can be seen within the framework of moderate anthropocentrism, as expressed in the ethics of responsibility. This view is based on the rational principle of evaluating the negative consequences of every human action toward the environment and future generations [76].

In the changing landscape of healthcare, the shift to patient-centered care represents a profound transformation that places MLPs at the center of efforts to improve patient outcomes, requiring not only technical expertise but also a broad and adaptable skill set capable of addressing the complexities of modern medical practice. This change requires MLPs to maintain exceptional analytical accuracy in diverse laboratory settings, ensuring that the reliability of test results supports accurate clinical decisions while elevating the quality of laboratory services by optimizing processes in the pre-analytical and post-analytical phases to improve both efficiency and overall effectiveness [77].

In addition, the role of MLPs goes beyond traditional laboratory functions, as they are increasingly charged with leveraging laboratory data to influence diagnostic and therapeutic pathways, which requires a deep understanding of the impact of such data on clinical outcomes and a commitment to integrate cutting-edge technologies and methodologies into routine practice. By engaging in collaborative research with clinicians, MLPs bridge the gap between laboratory innovation and its application in patient care, contributing to a healthcare model that seamlessly integrates advanced diagnostics with treatment strategies.

In addition, successful engagement with a variety of stakeholders requires strong relationship management skills, coupled with the ability to communicate effectively and tailor messages to different audiences. This emphasis on refined interpersonal and communication skills underscores a critical dimension of their responsibilities [78].

Ultimately, the changing paradigm of healthcare requires MLPs to adapt in tandem, developing a multifaceted skill set that includes analytical skills, process improvement, strategic use of laboratory data, active participation in collaborative research, stakeholder engagement, and effective communication. Together, these competencies form the basis for advancing the transition to a more patient-centered model of care [79].

The evolution toward patient-centered care marks a critical juncture in the advancement of skills within the realm of MLPs. Their role demands a spectrum of specific proficiencies. These include upholding analytical precision across diverse testing environments, enhancing the overall quality of laboratory services, and ensuring streamlined efficacy in pre-analytical and post-analytical phases. MLPs are also tasked with optimizing the utilization of laboratory data within diagnostic and treatment pathways and evaluating its impact on patient outcomes. Collaboration with clinicians in applied research becomes essential for seamlessly integrating novel technologies and methodologies into clinical practice. Moreover, adept relationship management with all stakeholders and honed communication skills are pivotal facets of their evolving role. In essence, MLPs are pivotal agents in the shift toward patient-centric care, necessitating a multifaceted skill set encompassing analytical expertise, process refinement, data utilization, collaborative research, stakeholder engagement, and effective communication [71,80,81,82,83]. The aspect of communication skills merits reflection. It may seem superfluous for professionals in a field that historically doesn’t directly interact with patients. However, this is not the case. The remarkable development of predictive medicine will lead to increasingly personalized prevention, demanding an even greater communicative effort from every healthcare professional. They will have the task of clearly delineating the meaning of genetic predisposition and the potential risk of illness as physicians increasingly have healthy patients and help them better manage their health capital.

The role of LMPs can extend beyond the field of communication. The recent COVID-19 pandemic has shown how difficult it can be for people to find reliable sources and accurate information when they are inundated with an excessive amount of information, often unreliable or misleading [84]. Such a flow of “misinformed” information can hinder efforts to effectively address public health crises. What do the sensitivity and specificity of a test mean? What are the consequences of low specificity? How does the rapid antigen test work, and when is it indicated? Is the molecular test more reliable? And serological tests? These were some of the questions that many sought answers to, even just by consulting the web. LMPs are also called to be protagonists in the health literacy process by providing information based on scientific evidence, transparent, correct, coherent in argumentation, clear and adequate to the understanding of the recipient, inclusive and reliable [85]. It is not just one of the many options but an ethical duty substantiated by criteria oriented toward the good of the recipient.

Finally, transparent and honest communication also applies to errors made in healthcare provided within laboratory activities [86]. This specific aspect of communication is an ethical and deontological duty that presupposes an open and honest relationship with the patient and their family. If errors and adverse events are communicated promptly, then the patient will be able to make informed choices, undergo appropriate treatments, and mitigate damages. Above all, it will strengthen the patient’s trust in healthcare workers and healthcare institutions.

## 4 Conclusion

LM plays a fundamental role in the healthcare ecosystem, closely collaborating with other medical sectors to provide accurate diagnoses, patient monitoring, and support for clinical decisions.

As highlighted, there are multiple ethical challenges associated with the practice of this medical activity that require special attention, such as privacy and data management, fair access to diagnostic technologies, result interpretation and communication, ethical use of genetic information, and potential conflicts of interest between profit pursuit and public health interest.

Addressing these challenges requires a balance between technological innovation and safeguarding rights, privacy, and equitable access to medical care. Adequate regulations, ethical guidelines, and increased public awareness can help mitigate these issues and ensure that LM is practiced ethically and responsibly. An additional ethical challenge lies in ensuring a balance between profit needs and public health priorities. It is essential for laboratories to adopt policies that prioritize patient well-being over economic gain, avoiding conflicts of interest by promoting transparent practices, ethical guidelines, and auditing mechanisms.

We believe that a possible response lies in adopting a well-structured ethical framework, such as that of the ethics of good work, which emphasizes integrity and excellence in professional practices for the benefit of individuals and society [87,88]. According to this ethical framework, the activity carried out by professionals working in the field of LM should be characterized by (a) an interdisciplinary co-design related to complexity theory and systemic thinking; (b) realistic knowledge that always starts from experience and seeks scientific truth as the basis for choices; (c) a management model useful for motivational engagement of all involved components; (d) awareness that every medical act is a free and responsible human act with intrinsic ethical value; (e) the retrieval of the political dimension of good work, that is, professional excellence as a tool in service to society and the common good; (f) the capacity for radical procedural innovation; and (g) placing the individual at the center of work, improving effectiveness and efficiency while ensuring sustainability [87]. These characteristics align with the personalist bioethical framework: interdisciplinarity reflects sociality, realistic knowledge aligns with wholeness, motivational management supports subsidiarity, and ethical awareness links with freedom and responsibility, professional excellence and innovation emphasizes sociality and responsibility, while patient-centeredness emphasizes human dignity, operationalizing the framework in LM. In pragmatic terms, it seems crucial to promote policies that encourage the adoption of clear bioethical frameworks and regulations regarding the use of AI technology and genetic data. Healthcare personnel should take part in training programs on bioethics in order to improve communicative and interpersonal skills to be able to handle complex situations. Finally, the development of diagnostic technologies that respect ethical principles and are accessible even in low-income settings should be encouraged.

We believe that by following this path, respectful and dignified treatment for all individuals can be ensured, promoting fair and universal access to safe and quality healthcare services.
